# Supplementary material for: Snow depth variability in the Northern Hemisphere mountains observed from space
Source: Nat Commun. 2019 Oct 11;10:4629. doi: 10.1038/s41467-019-12566-y (PMC6789005; doi:10.1038/s41467-019-12566-y)
Supplement: Supplementary file 2 — Reporting Summary [file 41467_2019_12566_MOESM2_ESM.pdf]

nature research

Corresponding author(s): Hans Lievens

Last updated by author(s): Sep 9, 2019

Reporting Summary

Nature Research wishes to improve the reproducibility of the work that we publish. This form provides structure for consistency and transparency in reporting. For further information on Nature Research policies, see Authors & Referees and the Editorial Policy Checklist.

Statistics

For all statistical analyses, confirm that the following items are present in the figure legend, table legend, main text, or Methods section.

n/a	Confirmed	
☐	☒	The exact sample size (*n*) for each experimental group/condition, given as a discrete number and unit of measurement
☐	☒	A statement on whether measurements were taken from distinct samples or whether the same sample was measured repeatedly
☒	☐	The statistical test(s) used AND whether they are one- or two-sided<br>*Only common tests should be described solely by name; describe more complex techniques in the Methods section.*
☒	☐	A description of all covariates tested
☒	☐	A description of any assumptions or corrections, such as tests of normality and adjustment for multiple comparisons
☐	☒	A full description of the statistical parameters including central tendency (e.g. means) or other basic estimates (e.g. regression coefficient) AND variation (e.g. standard deviation) or associated estimates of uncertainty (e.g. confidence intervals)
☐	☒	For null hypothesis testing, the test statistic (e.g. *F*, *t*, *r*) with confidence intervals, effect sizes, degrees of freedom and *P* value noted<br>*Give P values as exact values whenever suitable.*
☒	☐	For Bayesian analysis, information on the choice of priors and Markov chain Monte Carlo settings
☒	☐	For hierarchical and complex designs, identification of the appropriate level for tests and full reporting of outcomes
☐	☒	Estimates of effect sizes (e.g. Cohen's *d*, Pearson's *r*), indicating how they were calculated

Our web collection on statistics for biologists contains articles on many of the points above.

Software and code

Policy information about availability of computer code

Data collection	The Sentinel-1 data were pre-processed using the open source Google Earth Engine Python api. Further processing of the Sentinel-1 data was performed in Matlab R2018b
Data analysis	The analysis was performed in Matlab R2018b.

For manuscripts utilizing custom algorithms or software that are central to the research but not yet described in published literature, software must be made available to editors/reviewers. We strongly encourage code deposition in a community repository (e.g. GitHub). See the Nature Research guidelines for submitting code & software for further information.

Data

Policy information about availability of data

All manuscripts must include a data availability statement. This statement should provide the following information, where applicable:

- Accession codes, unique identifiers, or web links for publicly available datasets
- A list of figures that have associated raw data
- A description of any restrictions on data availability

The Sentinel-1 snow depth retrievals are available online at https://ees.kuleuven.be/project/c-snow. The source data for Figs. 1a, 4, 5, 7a,d correspond to the Sentinel-1 snow depth retrievals provided through the above-mentioned website. The source data underlying Figs. 7c,f and 8 are provided as a Source Data file.

Field-specific reporting

Please select the one below that is the best fit for your research. If you are not sure, read the appropriate sections before making your selection.

☐ Life sciences ☐ Behavioural & social sciences ☒ Ecological, evolutionary & environmental sciences

For a reference copy of the document with all sections, see nature.com/documents/nr-reporting-summary-flat.pdf

Ecological, evolutionary & environmental sciences study design

All studies must disclose on these points even when the disclosure is negative.

Study description	ESA and Copernicus Sentinel-1 satellite observations were used in a change detection algorithm to estimate the snow depth variability in the Northern Hemisphere mountains. The snow depth retrievals are (1) compared against a large collection of in situ snow depth measurements at 4175 locations, (2) compared with globally-available reanalysis data (for the months of February 2017 and February 2018) in terms of magnitude and elevation profile, and (3) used to estimate the total snow volume per mountain range in the Northern Hemisphere.
Research sample	Sentinel-1 data, used to produce the snow depth estimates, are freely available from various platforms, including the ESA and Copernicus open access hub (https://scihub.copernicus.eu/) and Google Earth Engine. We used the data provided by Google Earth Engine. The product used consists of ground-range detected (GRD) Interformetric Wide Swath (IW) backscatter observations in vertical-vertical and vertical-horizontal polarization, both from ascending and descending orbits.
Sampling strategy	All available Sentinel-1 observations were processed and included in the analysis.
Data collection	Data were pre-processed using Google Earth Engine's Python api, and further processed by the authors using Matlab 2018b.
Timing and spatial scale	The data were collected and processed between September 2016 through August 2018. The spatial resolution of the original Sentinel-1 data is 5 m by 20 m. The data were upscaled (by averaging in intensity domain) to 1 km². The spatial domain of the study corresponds to the Northern Hemisphere mountain ranges, defined by the Global Mountain Biodiversity Assessment (http://www.gmba.unibe.ch/). The temporal resolution of the Sentinel-1 data varies in space, depending on the latitude, the availability of ascending and/or descending orbit aquisitions, and the observation strategy outlined by ESA. Typically, an observation is available every 1 to 12 days, with more frequent data over Europe and near the poles.
Data exclusions	Sentinel-1 backscatter outliers were removed by excluding values that are 3 dB above the 90th-percentile or 3 dB below the 10th-percentile of the time series. The outlier removal is described in the paper and underwent peer review; pre-established criteria are not available.
Reproducibility	All attempts to repeat the experiment were succesful.
Randomization	The randomization is not relevant to our study, of which the objective is to evaluate global-scale retrievals of snow depth from satellite observations.
Blinding	The Sentinel-1 snow depth retrievals and corresponding in situ snow depth measurements were randomly sub-sampled in two groups of equal size, respectively for optimization and validation of the results.

Did the study involve field work? ☐ Yes ☒ No

Reporting for specific materials, systems and methods

We require information from authors about some types of materials, experimental systems and methods used in many studies. Here, indicate whether each material, system or method listed is relevant to your study. If you are not sure if a list item applies to your research, read the appropriate section before selecting a response.

Materials & experimental systems

n/a	Involved in the study	
☒	☐	Antibodies
☒	☐	Eukaryotic cell lines
☒	☐	Palaeontology
☒	☐	Animals and other organisms
☒	☐	Human research participants
☒	☐	Clinical data

Methods

n/a	Involved in the study	
☒	☐	ChIP-seq
☒	☐	Flow cytometry
☒	☐	MRI-based neuroimaging
